# *Fusarium* inhibition by wild populations of the medicinal plant *Salvia africana-lutea* L. linked to metabolomic profiling

**DOI:** 10.1186/1472-6882-14-99

**Published:** 2014-03-13

**Authors:** Mpumelelo M Nkomo, David DR Katerere, Hester HF Vismer, Thomas T Cruz, Stephane S Balayssac, Myriam M Malet-Martino, Nokwanda NP Makunga

**Affiliations:** 1 Department of Botany and Zoology, Stellenbosch University, Private Bag X1, Matieland, Stellenbosch 7602, South Africa; 2 PROMEC Unit, Medical Research Council (MRC), Tygerberg 7505, South Africa; 3 Groupe de RMN Biomédicale, Laboratoire SPCMIB (UMR CNRS 5068), Université Paul Sabatier, 118 route de Narbonne, 31062 Toulouse Cedex, France; 4 Research Associate, Institute for Plant Biotechnology, Department of Genetics, Stellenbosch University, Private Bag X1, Matieland, Stellenbosch 7602, South Africa; 5 Present Address: Department of Pharmaceutical Sciences, Tshwane University of Technology, Private Bag X680, Pretoria 0001, South Africa; 6 Present Address: Institute of Biomedical and Microbial Biotechnology, Cape Peninsula University of Technology, PO Box 1906, Bellville 7535, South Africa

**Keywords:** *Salvia africana-lutea*, Chemotypes, *Fusarium* species, Gas chromatography-mass spectrometry (GC-MS), Liquid chromatography-mass spectrometry (LC-MS), ^1^H nuclear magnetic resonance (NMR)

## Abstract

**Background:**

*Salvia africana-lutea* L., an important medicinal sage used in the Western Cape (South Africa), can be termed a ‘broad-spectrum remedy’ suggesting the presence of a multiplicity of bioactive metabolites. This study aimed at assessing wild *S. africana-lutea* populations for chemotypic variation and anti-*Fusarium* properties.

**Methods:**

Samples were collected from four wild growing population sites (Yzerfontein, Silwerstroomstrand, Koeberg and Brackenfell) and one garden growing location in Stellenbosch. Their antifungal activities against *Fusarium verticillioides* (strains: MRC 826 and MRC 8267) and *F. proliferatum* (strains: MRC 6908 and MRC 7140) that are aggressive mycotoxigenic phytopathogens were compared using an *in vitro* microdilution assay. To correlate antifungal activity to chemical profiles, three techniques *viz*. Gas chromatography-mass spectrometry (GC-MS); Liquid chromatography-mass spectrometry (LC-MS) and ^1^H Nuclear Magnetic Resonance (NMR) were employed. Principal Component Analysis (PCA) was applied to the NMR data. The partial least squares-discriminant analysis (PLS-DA) was used to integrate LC-MS and NMR data sets. All statistics were performed with the SIMCA-P + 12.0 software.

**Results:**

The dichloromethane:methanol (1:1; v/v) extracts of the plant species collected from Stellenbosch demonstrated the strongest inhibition of *F. verticillioides* and *F. proliferatum* with minimum inhibitory concentration (MIC) values of 0.031 mg ml^-1^ and 0.063 mg ml^-1^ respectively. GC-MS showed four compounds which were unique to the Stellenbosch extracts. By integrating LC-MS and ^1^H NMR analyses, large chemotype differences leading to samples grouping by site when a multivariate analysis was performed, suggested strong plant-environment interactions as factors influencing metabolite composition. Signals distinguishing the Stellenbosch profile were in the aromatic part of the ^1^H NMR spectra.

**Conclusions:**

This study shows the potential of chemotypes of *Salvia africana-lutea* in controlling fungal growth and consequently mycotoxin production. Products for use in the agricultural sector may be developed from such chemotypes.

## Background

Products of secondary metabolism are influenced by different environmental regions and plant-environment interactions [1,2]. Coupled with other factors such as genetic hybridization, studying wild populations for novel bioactives is of paramount importance. Studies of this nature may provide information that can be used in the improvement of the compositional quality of crops and medicinal plants that are utilized as phytomedicines.

Since the 1990s, metabolomics has been employed to hasten discovery of industrially useful chemicals [3] and also as a tool to provide deeper understanding of plant metabolism using systems biology approaches [4]. Since individual plant species have been known to manufacture over 100 000 secondary metabolites [5], robust techniques that are able to analyze these large metabolite numbers in the shortest time possible [3] are useful. The vast numbers also pose a problem in the identification of known and novel compounds. Nuclear magnetic resonance (NMR) is a convenient method for confirming the presence of known biomolecules and for assigning the chemical structure of novel ones. It can measure compounds in crude extracts or *in vivo* in a non-destructive nature [6]. There have been several NMR-based geographical characterizations of plant species, e.g. studies on *Coffea arabica*[7] and on *Withania somnifera*[8]. The advantage of using NMR is that no prior knowledge of the identity or quantity of the individual chemicals within the metabolomic pool is necessary. Therefore, the comparison of NMR profiles to differentiate groups is fast, convenient and a reproducible tool especially since the development of databases and software packages that can handle large datasets [9]. To consolidate NMR metabolite profiles, separation techniques such as gas chromatography and liquid chromatography hyphenated to mass spectrometry (GC-MS and LC-MS) can be utilized. Data generated from these techniques may yield additional information, providing a deeper view of a particular plant metabolome.

In this study, we focused on *Salvia africana-lutea* L. (Lamiaceae; commonly known as the beach or dune sage) which is one of 27 South African sages and 3 naturalized species [10]. In South Africa, the geographical distribution of this species is mainly limited to the south-western coastal area extending from the south-western part of the country through to the Cape Peninsula, and also, eastwards to the Eastern Cape Province in Port Alfred [11,12]. This grey-green aromatic woody shrub, growing to about 2 m, has leaves that store the essential oil in glandular trichomes and it has flowers that are easily recognizable in its reproductive phase (June to December). This plant displays mustard-yellow flowers that progressively become a burnt-orange colour as they senescence [12,13]. Studies on the phytochemical constituents of *Salvia* species originating from Africa has largely focused on profiling the compounds in the volatile essential oil fraction [14] but few studies have closely examined the non-volatile secondary metabolites.

This particular species is important to the ethnobotanical pharmacopoeia of the Western Cape because it is utilized for a myriad of disease symptoms such as coughs, sexual debility, mental and nervous conditions, throat inflammation, chronic bronchitis, tuberculosis, influenza, stomach ache, diarrhea, and urticaria, amongst others [14,15]. Biological properties reported mainly from *in vitro* experiments include: antibacterial, anti-inflammatory, anticancer and antioxidant activities [16-22]. Due to its phytomedicinal properties, commercialization and domestication has been proposed.

Ramogola [23] reported that extracts of *Salvia africana-lutea* inhibited *Fusarium* species. *Fusarium* infections may result in large agricultural production losses and potential contamination with mycotoxins, particularly in maize crops. This paper is a lead up from the study by Ramogola [23] and a more intensive metabolomic investigation of the species. We thus analyzed several different populations of *S. africana-lutea* to determine the extent of chemical variation. We also examined their efficacy against four strains of two *Fusarium* species. Biological activity is an expression of genotypic and phenotypic plasticity that leads to a changed secondary metabolite composition, often influenced by environmental perturbations [24]. This study aimed to assess the different metabolite profiles from the five populations and identify the most biologically active population, to link the activity with the elite chemotype.

## Methods

### Plant material

*Salvia africana-lutea* samples were collected from five different sites: Stellenbosch (S 33° 55.120′ E 18° 51.360′), Brackenfell Nature Reserve (S 33° 52.845′ E 18° 42.784′), Koeberg Nature Reserve (S 33° 40.128′ E 18° 26.524′), Silwerstroomstrand (S 33^o^ 34.632′ E 18^o^ 22.349′) and Yzerfontein (S 33° 22.309′ E 18° 10.871′). All locations are situated in the Western Cape Province of South Africa (Additional file 1). In total, 25 samples were collected from the different sites. Samples were identified by Dr Petra Wester and voucher specimens were deposited at the Stellenbosch University Herbarium. The arboreal plant parts were collected at two different times: April 2009 and June 2011. The plant material was oven-dried in closed brown bags at 50°C then ground to a fine powder using a mortar and pestle with liquid nitrogen prior to storage in the dark at room temperature.

### Extraction of plant material

For each site, extraction was carried out on the dried powdered aerial parts (5 g) with 20 ml of a 1:1 (v/v) methanol:dichloromethane mixture in a 60 ml glass test tube. These were then sonicated for 35 min (Bransonic 220, USA) before filtering with Whatman filter paper number 1. The extraction was repeated twice and pooled extracts were dried using a rotary evaporator (Buchi, Germany) at 55°C. Extracts were then stored in a desiccator prior to use. Micro-extraction was done on 0.5 g of dried powder using 10 ml of solvent mixture. These were then vortexed for 1 min and sonicated for 30 min. This step was repeated twice prior to centrifuging for 2 min at 4750 revolutions per minute (rpm). All extracts were filtered using cotton wool in a Pasteur pipette and collected in a 10 ml tube. Thereafter, they were evaporated to dryness *in vacuo*. Five extractions were performed for each site at one particular time. The experiments were repeated at least twice; unless otherwise stated.

### Fungal isolates and microtitre assays

Isolates of two fungal species *Fusarium verticillioides* (MRC 826 and 8267) and *F. proliferatum* (MRC 7140 and 6908) kept at the PROMEC Unit Culture Collection of the South African Medical Research Council (MRC) were used. The *Fusarium* isolates utilized are classified as high fumonisin B_1_ producers. Fungal isolates were grown on Carnation Leaf Agar (CLA) slants for 21 days at 25°C to induce spore production and stored in a cold room at 4°C prior to use. Fungal suspensions were prepared by dislodging the conidia in a 20 ml sterile 0.85% (w/v) saline solution. Conidia suspensions were standardized to a 0.5 McFarland concentration. The reference method for broth dilution antifungal susceptibility testing of filamentous fungi as described by the M38-A2 guide of the Clinical and Laboratory Standards Institute [25] was used to determine the minimum inhibitory concentration (MIC) for plant extracts. Each plant extract was resuspended in dimethyl sulfoxide (DMSO) to obtain stock solutions at a concentration of 50 mg ml^-1^. These were further diluted in the Roswell Park Memorial Institute RPMI-1640 medium at a 1:50 (v/v) ratio to obtain final concentrations of 1.0, 0.5, 0.25, 0.125, 0.063, 0.031, 0.016, 0.008, 0.004 and 0.002 mg ml^-1^ in the 10 wells. Voriconazole (Vfend®, Pfizer) was used as a positive control. A row of DMSO and medium was used as a solvent control, while the last negative control had the medium only (growth control).

### LC-MS analysis

Five extracts from the different study sites were resuspended in 1 ml of a 50% (v/v) mixture of acetonitrile and H_2_O containing 0.1% (v/v) formic acid. The suspensions were vortexed for 1 min then sonicated for 5 min, vortexed again for 1 min prior to spinning at 10 000 rpm for 10 min. The supernatant (3 μl) was injected into the LC-MS instrument. Metabolites were separated using a gradient of H_2_O with 0.1% formic acid (solvent A) and acetonitrile (solvent B), using a Waters UPLC at a flow rate of 0.4 ml min^-1^ on a Waters BEH C18, 2.1×50 mm column. Mass spectrometry was obtained on a Waters SYNAPT™ G2 MS (Manchester, England) using electron spray ionization (ESI) running in positive mode with a cone voltage of 15 V. The injections were repeated once to ensure repeatability.

### NMR analysis

Twenty one dried micro-extracts from five different locations (four from Stellenbosch, four from Yzerfontein, three from Silwerstroomstrand, five from Koeberg and five from Brackenfell) were vortexed for 15 s after the addition of 2.5 ml of DMSO-d6 (Eurisotop, France). The mixture was filtered and 550 μl of the filtrate were analyzed. A 10 mM solution of sodium 2,2,3,3-tetradeutero-3-trimethylsilylpropionate (TSP) (Sigma-Aldrich, St. Louis MO, USA) (10 μl) was added as an internal chemical shift reference before the NMR analysis. One dimensional (1D) ^1^H NMR spectra were recorded at 298 K on a Bruker Avance 500 NMR instrument operating at 500.13 MHz, equipped with a 5-mm TCI cryoprobe. The ^1^H NMR experiments were acquired using a relaxation delay-pulse-acquisition sequence. Acquisition parameters were as follows: pulse width of 2.8 μs (flip angle ≈ 30°), relaxation delay of 4 s, 64 K data points, spectral width of 9500 Hz (19 ppm) and 128 scans. All FIDs were processed using the Bruker TopSpin 2.1 software with one level of zero-filling and a line broadening of 0.7 Hz. Baseline correction was performed on each spectrum and spectra were referenced to the signal of TSP at δ 0.00 ppm.

### Chemometric analysis of the data

The 1D ^1^H NMR spectra were transferred to the KnowItAll® software (Bio-Rad, USA). The bin area method was used to segment the spectra between 0 and 13.1 ppm with the variable size intelligent bucketing tool included in the KnowItAll® package. Bucket sizes ranged from 0.01 to 0.30 ppm. The spectral regions containing the NMR signals of DMSO (δ 2.47-2.57 ppm) and its ^13^C satellites (δ 2.36-2.40 ppm, and 2.63-2.67 ppm), methanol (δ 3.17-3.20 ppm), H_2_O (δ 3.33-3.45 ppm) and dichloromethane (δ 5.75-5.80 ppm) were removed. A manual filtering procedure was applied to the whole spectrum to exclude buckets that contained only noise. A total of 119 variables were considered for subsequent statistical analyses. For spectrum normalization, integrated regions were divided by the total area of the spectrum and multiplied by the mean value of the corresponding family previously calculated. Data were preprocessed by mean-centering. The unit variance (UV) scaling method was applied prior to analysis.

Both principal component analysis (PCA) and partial least squares-discriminant analysis (PLS-DA) were performed with the SIMCA-P + 12.0 software (Umetrics, Umeå, Sweden) and for the t-tests, the R software (R Development Core Team, 2012) was used. The predictive ability of the PLS-DA models was assessed from the values of Q^2^_cum_ (> 0.5), R^2^Y_cum_ (> 0.7) and R^2^X_cum_ parameters. The statistical significance of R^2^Y and Q^2^ parameters was also estimated through the response permutation test where the Y matrix was 999 times randomly permuted when the X matrix was fixed [26]. For determining the discriminating variables between classes, loading plots, coefficient plots, variable importance in the projection (VIP) from PLS-DA models, and p-values (<10^-3^) of the t-tests on the variables arising from the coefficient plot and VIP were considered. Several PLS-DA models were built: (i) from variables of the whole ^1^H NMR spectrum (119); (ii) from variables of the 13-5 ppm region of the ^1^H NMR spectrum (69); and, (iii) from a combination of the 69 variables of the 13-5 ppm region of the ^1^H NMR spectrum with the 39 variables corresponding to the main peaks of the LC-MS chromatograms.

### GC-MS analysis

For each sample 100 mg of ground plant material was utilized. A similar protocol to that used by Glassop et al. [27] was employed with only a minor change for the derivatization of solvent extracts. Myo-inositol (2 mg ml^-1^) was added to the ground plant material then dissolved in 350 μl of methanol:chloroform (1:1, v/v) in a 2 ml microcentrifuge tube. All sample tubes were placed in a sonicator (Bransonic 220, USA) at room temperature for 45 min. Samples were then centrifuged at 1 200 rpm for 10 min at room temperature in a centrifuge (Biofuge pico, Germany). Ribitol (Sigma-Aldrich; Germany) was included as an internal standard after derivatization. One μl of the samples was injected for a splitless run with an initial temperature of 70°C (5 min) and a maximum oven temperature of 330°C (equilibration time of 0.25 min) was used. Analysis was performed using a network GC system (6890 N) coupled to inert XL EI/CI Mass Selective Detector (MSD) 5975B (Agilent Technologies Inc., Palo Alto, CA) equipped with a CTC Analytics PAL autosampler had. Separation was achieved with a capillary column (Restek RTX200; trifluoropropylmethyl (30 m in length; 250 μm diameter; 1 μm in thickness)).

The temperature was increased from 76°C (1 ramp min^-1^) to 320°C (4 ramp min^-1^) The run time was 72 min and helium gas was used as a carrier at a flow rate of 53.7 ml min^-1^. The instrument was set to the following conditions: pressure of 62.6 kPa, purge flow of 50 ml min^-1^ for 2 min, flow rate of 1 ml min^-1^ (37 cm sec^-1^) and a data rate of 20 Hz.

The mass spectrometer was operated in electron ionization (EI) mode at ionization energy of 70 eV, scanning from 35 to 600 m/z in positive mode. Caffeic acid, rosmarinic acid, myo-inositol, glucose, galactose and mannose were used as standards to aid with identification of constituents in the extracts. All standards were purchased from Sigma-Aldrich (Germany) except for the mannose which was provided by Merck (Germany). Data were analyzed using the MSD Chemstation software which was linked to the National Institute of Standards and Technology (NIST) mass spectral search program library ver. 2.0 d (2005; standard reference data program of the National Institute of Standards and Technology, USA) for peak identification of metabolites. A library match of 80% and above for metabolites from the NIST library were regarded as likely hits. The relative abundance of metabolites was recorded using the total ion chromatogram peak integration (Additional file 2).

## Results and discussion

All test plant samples showed good *in vitro* antifungal activity against the test strains of *Fusarium*, with MIC values between 0.031 mg ml^-1^ and 0.5 mg ml^-1^ (Table 1). Although there is no congruency in terms of the classifications used for antifungal *in vitro* assays, several authors [28,29] have suggested that MIC values below 0.5 mg ml^-1^ should be regarded as representing phytochemical extracts with strong inhibition and above 1.6 mg ml^-1^ are regarded as weak inhibitors. The Stellenbosch site extract was the most active against the tested fungal strains for the two years (2009 and 2011). It showed the best activity against *F. verticillioides* MRC 8267 and MRC 826 at 0.031 mg ml^-1^ (Table 1). This activity compared favourably with the positive control, voriconazole (MIC - 0.0156 mg ml^-1^) (Table 1). Our data was similar to that of Ramogola [23] where the strongest inhibition against *F. verticillioides* MRC 8267 (MIC - 0.02 mg ml^-1^) was reported for extracts produced from Stellenbosch plants. In this study, the weakest activity against *F. proliferatum* (MRC 6908) at 0.5 mg ml^-1^ was exhibited by the extract from the Yzerfontein population but this still falls into the “strong inhibitor” category according to the recommendations by Souza et al. [29]. Differences in the MIC values were also associated with years of harvesting. Thus plant material harvested in 2011 was generally more potent than that collected in 2009 (Table 1). This is likely to be as a result of chemical decomposition due to prolonged storage [30]. This result is not surprising as Kamatou et al. [20] demonstrated the correlation of seasonal variation on the essential oil composition and biological activity of *S. africana-lutea*.

**Table 1:** Minimum inhibitory concentrations (MIC) values observed after 48 h from crude plant extracts obtained from the five study sites

	**Collection year**	***F. proliferatum***		***F. verticillioides***	
**Extract/Isolate**		**MRC 6908**	**MRC 7140**	**MRC 8267**	**MRC 826**
Stellenbosch	2009	0.125	0.063	0.125	0.125
	2011	0.125	0.125	0.031	0.031
Brackenfell	2009	0.25	0.125	0.125	0.25
	2011	0.25	0.25	0.125	0.25
Koeberg	2009	0.25	0.25	0.125	0.25
	2011	0.25	0.25	0.25	0.25
Silwerstroomstrand	2009	0.25	0.125	0.125	0.25
	2011	0.25	0.25	0.063	0.125
Yzerfontein	2009	0.125	0.25	0.125	0.25
	2011	0.50	0.25	0.063	0.125
Voriconazole		0.0156	0.0156	0.0156	0.0156

MIC results observed after 48 h are regarded as: strong inhibitors (MIC < 0.5 mg ml^-1^); moderate inhibitors (0.6 ≤ MIC ≤ 1.5 mg ml^-1^) and weak inhibitors (MIC < 1.6 mg ml^-1^) [29].

Seasonal-climatic influences may thus cause great changes in the phytochemical profiles of these plants. In the present study the impact of temporal variation was compared to assess differences between the 2 years in terms of anti-fungal actions as well as chemical constituents bioactivity.

**Figure 1:**
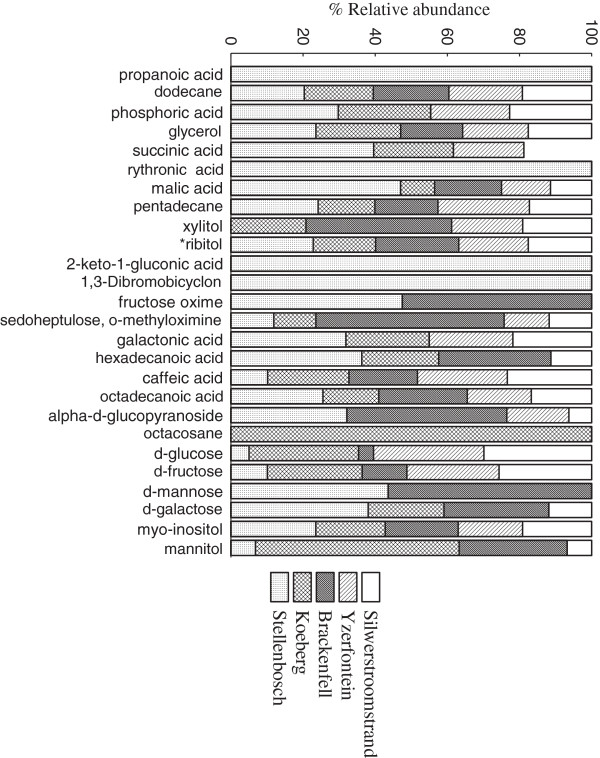
Distribution of compounds detected using GC-MS across sites.

In assessing the chemotype variations, we first analyzed the extracts using GC-MS. Compounds detected were mostly primary metabolites such as monosaccharides, organic acids and fatty acids. This was expected as the derivatization protocol favored the extraction of these compounds. Figure 1 displays the distribution of some of the compounds in the sampled sites using an 80% identification limit, while Table 2 shows the retention times and Kovats indices. Interestingly, those samples extracted from Stellenbosch exhibited several remarkable differences which discriminated them from the other locations. They contained propanoic acid, rythronic acid, 2-keto-1-gluconic acid and 1,3-dibromobicyclon not detected in the other locations. In contrast, some compounds that were common to all the other samples, such as xylitol, were not observed in the Stellenbosch samples. These compounds may not be directly linked to the difference in antifungal activity from the other sites, but serve to highlight metabolic signatures that distinguish the Stellenbosch samples from the others.

**Table 2:** Kovats indices of compounds identified using GC-MS

**Compound**	**Retention time (min)**	**Kovats index**
**Propanoic acid**	12.847	1057
**Dodecane**	19.403	1214
**Phosphoric acid**	22.188	894
**Glycerol**	22.386	940
**Succinic acid**	23.836	1132
**Malic acid**	29.751	1294
**Pentadecane**	30.149	1500
**Rythronic acid**	31.958	1518
**Xylitol**	36.487	1491
**Ribitol**	36.641	1491
**2-keto-1-gluconic acid**	37.616	1748
**1,3-dibromobicyclon**	38.996	1674
**D-fructose**	40.452	1726
**Fructose oxime**	40.709	2323
**D-glucose**	40.888	1698
**D-mannose**	41.074	1698
**D-galactose**	41.408	1698
**Sedoheptulose, o-methyloxime**	41.812	2533
**Galactonic acid**	43.403	1991
**Hexadecanoic acid**	44.737	2047
**Myo-inositol**	45.706	2152
**Mannitol**	46.559	1752
**Caffeic acid**	46.681	1985
**Octadecanoic acid**	49.138	2238
**Alpha-D-glucopyranoside**	56.798	3552
**Octacosane**	59.922	2800

Chemicals were identified using the NIST library and ribitol was used as the internal standard.

**Figure 2:**
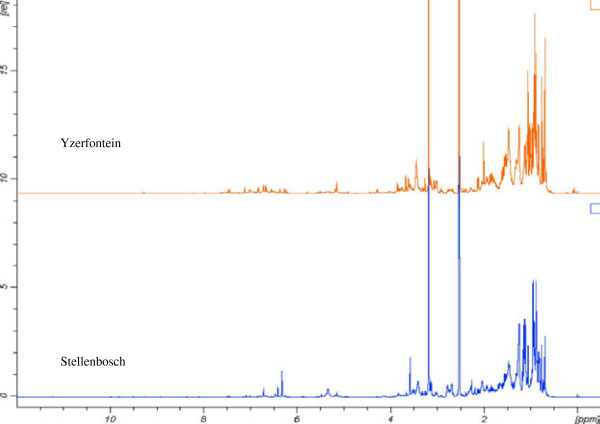
^**1**^**H NMR spectra of the two most different sample sites.**

**Figure 3:**
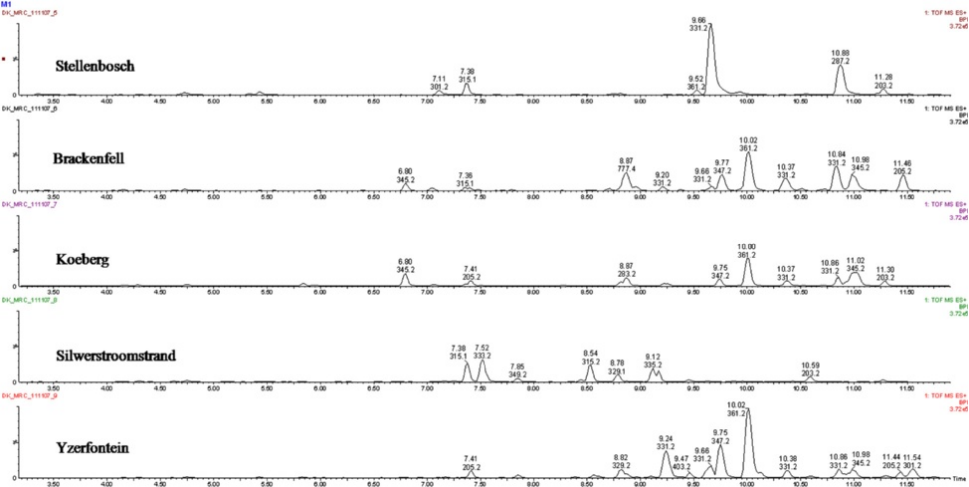
LC-MS spectra of the five different sample sites.

**Figure 4:**
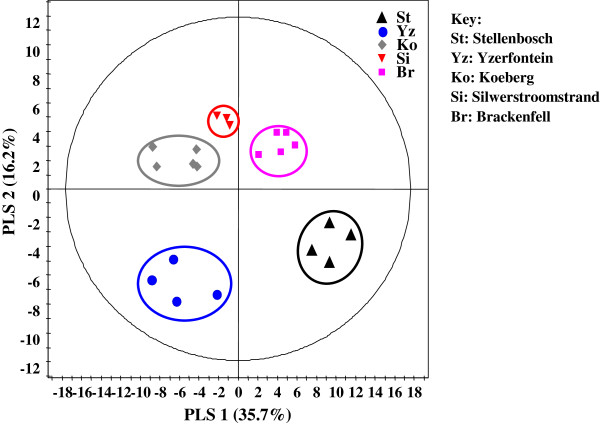
**Score plots of the PCA (A) (6 axes, R**^**2**^**X 0.995) and PLS-DA (B) (6 axes, Q**^**2**^_**cum **_**0.89, R**^**2**^**X**_**cum **_**0.88, R**^**2**^**Y**_**cum **_**0.97) on **^**1**^**H NMR data (entire spectrum) of samples from the 5 sites.**

**Figure 5:**
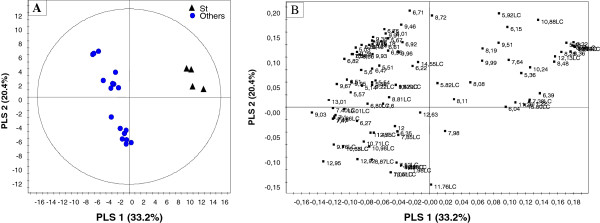
**Score plot (A) and loading plot (B) of the PLS-DA on **^**1**^**H NMR (13-5 ppm region) and LC-MS data of samples from Stellenbosch (denoted as St) versus other sites (validation parameters of the model: 3 axes, Q**^**2**^_**cum **_**0.96, R**^**2**^**X**_**cum **_**0.60, R**^**2**^**Y**_**cum **_**0.99).**

For in-depth metabolite profiling especially on secondary metabolites, ^1^H NMR and LC-MS were performed (Figures 2 and 3). Chemical profiles were highly complex especially with NMR. All populations showed differences with both techniques presented for instance on the score plots of the PCA and PLS-DA of ^1^H NMR data where 4 and 5 clusters could be observed respectively (Figure 4). Plants from Stellenbosch are subjected to constant perturbations as they are in close proximity to human dwellings while the other locations are in more protected areas with less interaction. It has been reported that accumulation of aromatic compounds, mainly phenylpropanoids, flavonoids and other such metabolites, which have high antimicrobial action, becomes favoured when plants are under stress [1]. We thus performed a PLS-DA on the 69 variables of the 13-5 ppm region of the ^1^H NMR spectra of Stellenbosch samples versus all other samples. The main signals distinguishing the Stellenbosch profile from the others were located at 8.36, 8.28, 7.79, 6.32 and 5.48 ppm (Figure 5). On combining ^1^H NMR (69) and LC-MS (39) variables, the separation was driven mainly by the same NMR signals along with five LC peaks with retention times of 7.11, 9.66, 9.93, 10.88 and 12.13 min. We therefore can tentatively state that the Stellenbosch site is markedly different from other sites both in chemical composition and biological (antifungal) activity. The challenge thus far is in linking the discriminating peaks to bioactivity. Work is in progress to determine the structure of these compounds, which might be at least for some of them, flavonoids, and to further confirm the antifungal activity of these pure compounds.

## Conclusions

*Salvia africana-lutea* extracts have strong anti-*Fusarium* properties and this activity holds potential for product development. This may be of particular interest to manufacturers of agrochemicals. It becomes imperative to follow this study with one that will rigorously correlate the chemical differences to bioactivity in an effort to identify the metabolites responsible for it. Apart from this, a deeper understanding of the chemical constituents which compose the Stellenbosch extracts will be beneficial as part of a commercial and domestication platform for *S. africana-lutea.* Indeed, plant extracts that possess such antimicrobial qualities show great potential for development into chemotherapeutic or preventive drugs and might ultimately replace the current choices at our disposal especially because many of the antifungal agents available in the market may become redundant as drug tolerance is developed by microorganisms.

## Abbreviations

CLA: Carnation leaf agar; DMSO: Dimethyl sulfoxide; ESI: Electron spray ionization; MIC: Minimum inhibitory concentration; MRC: Medical Research Council; NIST: National Institute of Standards and Technology; rpm: Revolutions per minute; TSP: Sodium 2,2,3,3-tetradeutero-3-trimethylsilylpropionate; UV: Unit variance; VIP: Variable importance in the projection.

## Competing interests

The authors declare that they have no competing interests.

## Authors’ contributions

MN and NPM contributed with the conception of the study and GC-MS analysis. MN, NPM, DK and HV were responsible for the design and execution of the antifungal assays and the experiments were conducted in the laboratories of HV. DK and MN were involved in the LC-MS analysis. TC, SB and MMM as a group carried out the NMR and chemometric analysis of LC-MS and NMR All authors contributed to further writing of the manuscript. All authors read and approved of the final manuscript.

## Pre-publication history

The pre-publication history for this paper can be accessed here:

http://www.biomedcentral.com/1472-6882/14/99/prepub

## Supplementary Material

Additional file 1 Distribution of Salvia africana-lutea populations along the coastal regions of South Africa. Click here for file

Additional file 2 Total ion chromatogram peak integration of gas chromatography-mass spectrometry. Click here for file
