# Supplementary figures and images for: Fusarium inhibition by wild populations of the medicinal plant Salvia africana-lutea L. linked to metabolomic profiling
Source: BMC Complement Altern Med. 2014 Mar 13;14:99. doi: 10.1186/1472-6882-14-99 (PMC4101822; doi:10.1186/1472-6882-14-99)

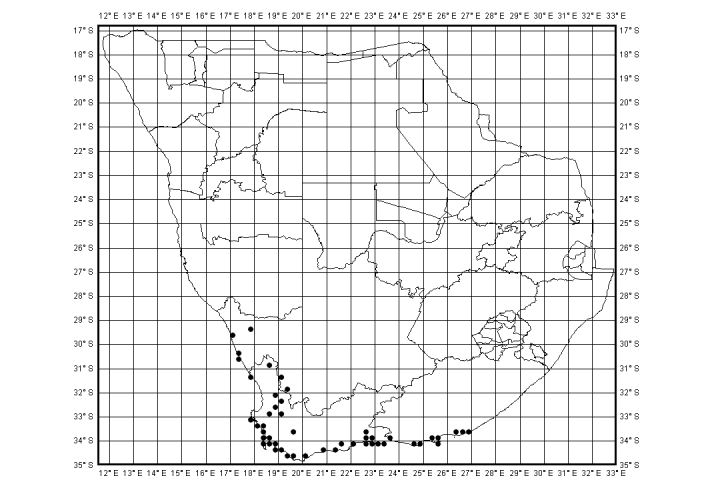


Distribution of Salvia africana-lutea populations along the coastal regions of South Africa.

Supplement: Additional file 1 — Distribution of Salvia africana-lutea populations along the coastal regions of South Africa. [file 1472-6882-14-99-S1.docx]

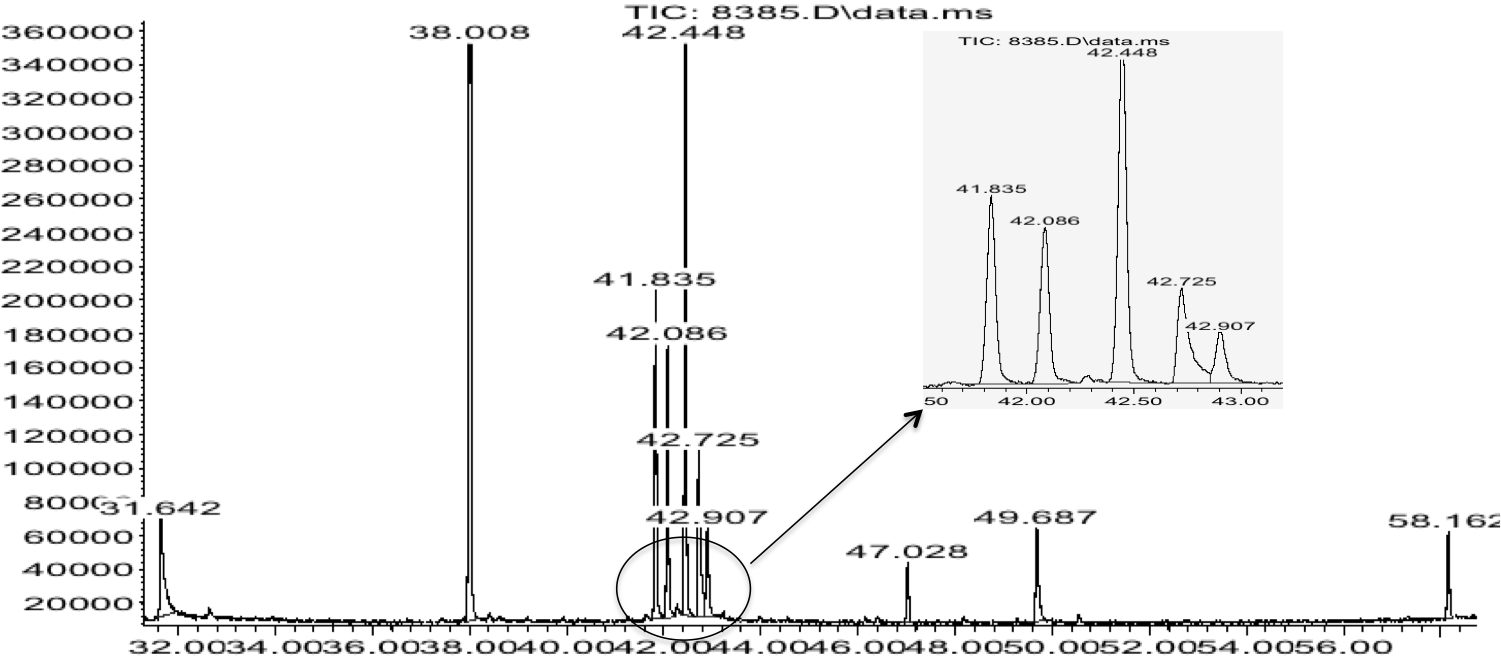

Supplement: Additional file 2 — Total ion chromatogram peak integration of gas chromatography-mass spectrometry. [file 1472-6882-14-99-S2.png]
